# Effects of person-vocation fit and core self-evaluation on career commitment of medical university students: the mediator roles of anxiety and career satisfaction

**DOI:** 10.1186/1752-4458-8-8

**Published:** 2014-02-20

**Authors:** Wei Xiao, Liang Zhou, Qing Wu, Yan Zhang, Danmin Miao, Jiaxi Zhang, Jiaxi Peng

**Affiliations:** 1 Department of Psychology, Fourth Military Medical University, Chang Le Western Street No.169, Xi’an, Shanxi Co. 710032, China; 2 Foreign Language Teaching and Researching Office of Basic Education Department, Chongqing Communication Institute, Chongqing, China

**Keywords:** Medical university student, Person-vocation fit, Core self-evaluation, Career commitment

## Abstract

**Background:**

How the career commitment of medical university students can be improved is an underinvestigated topic.

**Aim:**

This experimental study aims to explore the factors that influence career commitment of medical university students.

**Methods:**

One hundred eighty-two medical university students completed the vocational value questionnaire, state anxiety scale, core self-evaluation scale, Minnesota satisfaction questionnaire, and the Chinese career commitment questionnaire.

**Results:**

(1) A mismatch was found between the vocational value and the medical career of medical university students, primarily in their self-development; (2) Core self-evaluation can significantly predict the continued commitment of medical university students; (3) Vocational value, career fit, and core self-evaluation can significantly predict the affective commitment and normative commitment of medical university students, while state anxiety and vocational satisfaction play significant mediating roles.

**Conclusions:**

Both person–vocation fit and core self-evaluation can affect the career commitment of medical university students, while job satisfaction and state anxiety play mediating roles.

## Background

Medical university students in China are the future doctors and nurses of the country. The adherence of doctors and nurses to their difficult, dangerous professions depends on their commitment to their organizations and careers. Previous studies have proven that person–organization fit can significantly predict organizational commitment and career satisfaction, and other studies have begun to search for similar ties among person–vocation fit, career commitment, and career satisfaction. Both person–organization fit and person–vocation fit represent different levels of person–environment fit. Kristoff and Cable identified three aspects of person–environment fit, namely, consistency fit, which could be observed when two parties bear similar basic features; supply–demand fit, which refer to satisfaction of personal demands by the environment; and requirement–capacity fit, which could be observed when individuals have the capacities required by the environment [1,2]. Previous studies on person–vocation fit have mainly focused on career selection theories and have encouraged individuals to choose their careers based on their interests. For instance, Holland found that both individuals and their careers possess unique characteristics and argued that person–vocation fit aims to ensure a match between the career of an individual and his or her characteristics. However, the fit between the characteristics and the career of an individual actually refers to consistency fit, which is merely a part of person–vocation fit. The concept of “vocational value–career fit” that is introduced in this study is also a part of the person–vocation fit. Vocational values represent the expectation of individuals as well as the importance of evaluating the results of their work. Gaps exist between the expectations and realities of individuals. The expected work results, such as the development of opportunities and favorable working environments, are usually not satisfied by the careers of individuals. Therefore, absence of fit between expectation and vocation is commonly observed. Extant studies indicate that vocational value–career fit can significantly predict the career satisfaction of individuals [3]. Core self-evaluation (CSE) represents the stable personality trait that encompasses the subconscious of individuals as well as their fundamental evaluations about themselves, their abilities, and their control. CSE was introduced by Judge, Locke, and Durham, who defined such concept as the higher-order factor of four personality dimensions, namely, locus of control, neuroticism, generalized self-efficacy, and self-esteem [4,5]. Subsequent studies found that CSE is positively related to the salary, career commitment, and capacity of individuals to cope with organizational changes [5].

The relationship among satisfaction, pressure, and commitment has always been a hot topic among scholars. However, extant studies have failed to determine if pressure and work satisfaction affect commitment or if commitment affects work satisfaction and pressure. In 1989, Mathieu found that satisfaction preceded commitment [6], whereas Amy and Chockalingam found that career commitment preceded career satisfaction [7]. Glazer and Kruse conducted a survey on 506 Israeli nurses in 2008 and found that job-targeted anxiety could predict resigning trends, whereas organizational commitment played a significant mediating role during the process [8].

## Objectives

Based on previous studies, we have identified several relationships among five variables, namely, fitness, satisfaction, CSE, pressure, and commitment. Fitness affects satisfaction and pressure, CSE can predict satisfaction and commitment, and satisfaction and pressure may affect commitment. Similar relationships may exist among person–vocation fit, career satisfaction, career pressure, career commitment, and CSE at the career level. This research will replace person–vocation fit with vocational–value fit and will replace career pressure with career-targeted anxiety to investigate and verify the relationship mechanism among the five abovementioned variables.

## Materials and methods

### Participants

The survey selected 182 university students from three medical universities in Xi’an. Out of the 182 distributed questionnaires, 180 were returned by the respondents, 167 of which were valid, which indicates a response rate of 92.4%. Of these 167 valid responses, 107 participants were men and 60 participants were women. The proportions of first year, second year, third year, and fourth year medical university students were 29.9%, 24.4%, 27.5%, and 22.2%, respectively.

The survey data were collected in Xi’an, China from July 17^th^ to July 18^th^. All participants provided written informed consent before participating in the survey. This research satisfied the ethical guidelines of the Northwest University and was approved by the university ethics committee (No. 20130221). The research also adhered to the legal requirements of the People’s Republic of China.

### Material

#### Vocational value questionnaire

The 22-item vocational value questionnaire covered three dimensions [9], namely, reputation and status, basic security, and self-development. The participants were required to evaluate the importance of each item, including “reputation” and “life with family,” as well as to evaluate the possibilities for their careers to satisfy these items. Their evaluations were then used to calculate two variables, namely, the vocational values of these individuals and the possibility for their medical careers to satisfy such values. Previous studies that used exploratory and confirmatory factor analyses showed that the scale was composed of three dimensions. The half reliability was 0.810, the loading of the items was between 0.38 and 0.65, and the fitting precisions of cross validity and convergent validity are both at acceptable levels [9]. In the current study, the Cronbach’s alpha coefficient for the whole scale was 0.738.

#### State anxiety scale

The state anxiety scale was introduced by Spiel in 1997 [10]. This study mainly examines the state anxiety that is caused by careers. The following instruction was given at the beginning of the state anxiety questionnaire: “Please focus on your future career and choose the answers that are most in line with your current experience. Your answers are neither correct nor incorrect. Therefore, please don’t think too much about the questions. Just select the answers that are most in line with your current feelings.” In this study, the Cronbach’s alpha coefficient for the state anxiety scale was 0.850.

#### Core self-evaluation scale

The core self-evaluation scale (CSES) of Judge et al. [11] is a 12-item self-report measure of CSE. These items are rated from 1 (strongly disagree) to 5 (strongly agree). Examples of items include “I am confident that I will achieve the success that I deserve in this life”’ and “I feel worthless sometimes when I fail.” The scores are the sum of items that were reverse coded from the relevant items. Previous studies that used exploratory and confirmatory factor analyses found that the scale was composed of a single dimension. The half reliability was 0.769, the loading of the items was between 0.42 and 0.70, and high fitting precisions of cross validity and convergent validity were found [11]. In the current study, the Cronbach’s alpha coefficient for CSES was 0.774.

#### Minnesota satisfaction questionnaire

The Minnesota satisfaction questionnaire (MSQ) of Weiss et al. [12] is a 20-item self-report measure of job satisfaction. The items are rated from 1 (strongly dissatisfied) to 5 (strongly satisfied). The total scores range from 20 (low job satisfaction) to 100 (high job satisfaction). The items include “the chance to try out some of my own ideas.” In this study, the Cronbach’s alpha coefficient for MSQ is 0.893.

#### Chinese career commitment questionnaire

The Chinese career commitment scale comprises 16 items that are rated from 1 (strongly disagree) to 5 (strongly agree). These items include “I am proud of my occupation” and “Everyone must be loyal to his or her occupation, so I must not change my job easily.” This scale has been widely used among Chinese populations for its acceptable validity and reliability. Previous studies that used exploratory and confirmatory factor analyses showed that the scale was composed of three dimensions [13]. The re-test reliability was 0.74, the loading of the items was between 0.37 and 0.74, and the fitting precisions of both cross validity and convergent validity are high. In this study, the Cronbach’s alpha coefficients were 0.923, 0.703, and 0.798 for the three sub-scales.

Data analyses were performed by using the SPSS statistical software package. Paired t-test, correlation analysis, one-way ANOVA, and regression analysis were conducted in the analyses. A P-value < 0.05 was considered statistically significant. The path analysis was performed by using AMOS 17.0.

## Result

### Vocational value–career fit of medical university students

Table 1 shows the vocational value–career fit of the participants. The personal demands of the participants were significantly higher than the possibilities that were provided by their careers, which reflected the unfitness of their vocational value–career. Following the general approach for indirect test of fitness, the demands of the individuals were subtracted from the possibilities that were provided by their careers.

**Table 1:** **Vocational value-career fit (*n*** **= 167)**

	**Demands**	**Possibility**	***t***
Reputation and status	3.179 ± 0.63	2.91 ± 0.79	4.659^***^
Self development	3.805 ± 0.60	3.10 ± 0.70	12.181^***^
Basic security	3.80 ± 0.71	3.41 ± 0.56	6.500^***^

Note: ***, *p* < 0.001.

Table 2 shows the differences between the demands of individuals and the possibilities that their careers offer in different dimensions. Tables 1 and 2 show unfitness between the demands and the supplies of medical university students. Such unfitness was mainly manifested in the individual development dimension.

**Table 2:** **Three dimensions of demands-supplies differences (*n*** **= 167)**

**Reputation unfitness**	**Basic security unfitness**	**Development unfitness**	***F***
0.27 ± 0.745	0.387 ± 0.76	0.72 ± 0.77	24.416^***^

Note: ***, *p* < 0.001.

### Correlative analysis

Table 3 shows close, positive correlations among the three dimensions of vocational value–career fit. These three dimensions are positively related to state anxiety and affective commitment. Correlations were also found among CSE and all other variables, except for reputation unfitness. CSE was the only variable that was correlated with cost commitment. State anxiety was negatively correlated with satisfaction, affective commitment, and nominative commitment, whereas satisfaction was closely correlated with all variables except for reputation unfitness and cost commitment.

**Table 3:** **Descriptive statistics and correlation analysis of variables (*n*** **= 167)**

	**1**	**2**	**3**	**4**	**5**	**6**	**7**	**8**	**9**
1. Reputation unfitness		0.227^**^	0.175^*^	0.179^*^	−0.015	−0.07	−0.155^*^	0	−0.143
2. Basic security unfitness			0.575^***^	0.351^***^	−0.357^***^	−0.227^**^	−0.402^***^	0.005	−0.408^***^
3. Development unfitness				0.385^***^	−0.404^***^	−0.181^*^	−0.527^***^	−0.082	−0.495^***^
4. State anxiety					−0.629^***^	−0.518^***^	−0.583^***^	0.148	−0.465^***^
5. Career satisfaction						0.424^***^	0.672^**^	−0.001	0.543^**^
6. Core self evaluations							0.381^***^	−0.312^***^	0.337^**^
7. Affective commitment								0.037	0.740^***^
8. Cost commitment									0.118
9. Normative commitment									
Mean	0.27	0.38	0.72	2.50	3.54	3.11	3.65	2.91	3.80
SD	0.74	0.76	0.77	0.44	0.46	0.50	0.80	0.67	0.67

Note: *, *p* < 0.05; **, *p* < 0.01; ***, *p* < 0.001.

### Mediation test of state anxiety and career satisfaction

By following the mediation test rules that were introduced by Baron and Kenny in 1986 [14], we tested the intermediary roles of state anxiety and career satisfaction among CSE, vocational value–career fit, and career commitment.

Table 4 shows that all regression models are significant. From models 2, 6, and 7, the regression coefficient of development unfitness to affective commitment was −0.424 when the former was used as an independent variable. The regression coefficient decreased to −0.307 and −0.249 when state anxiety and career satisfaction were introduced, respectively. Therefore, both state anxiety and career satisfaction partly mediated the effect of person–vocation fit to emotional career. From models 3, 8, and 9, the regression coefficient of CSE to affective commitment was 0.277 (p < 0.001) when the former was used as the independent variable. The regression coefficient became insignificant after state anxiety and career satisfaction were introduced, which indicates that the prediction of affective commitment by CSE was completely mediated by state anxiety and career satisfaction.

**Table 4:** Stepwise regression of affective commitment

	**Model 1**	**Model 2**	**Model 3**	**Model 4**	**Model 5**	**Model 6**	**Model 7**	**Model 8**	**Model 9**
Personal variables									
Development unfitness		−0.424^***^				−0.307^***^	−0.249^***^		
Core self evaluations			0.277^***^					0.069	1.137
Anxiety				−0.503^***^		−0.408^***^		−0.469^***^	
Career satisfaction					0.591^***^		0.509^***^		8.958^***^
*R*^*2*^	0.174^***^	0.37^***^	0.24^***^	0.379^***^	0.495^***^	0.452^***^	0.541^***^	0.378^***^	0.496^***^
*△R*^*2*^	0.174^***^	0.151^***^	0.066^***^	0.197^***^	0.306^***^	0.269^***^	0.352^***^	0.200^***^	0.310^***^

Note: ***, *p* < 0.001.

We tested the mediating effects of both state anxiety and career satisfaction among person–vocation fit, CSE, and normative commitment by using the same approach; the results are shown in Table 5. All the regression models were significant. From models 2, 6 and 7, the regression coefficient of development unfitness to normative commitment was −0.416 when the former was used as the independent variable. The regression coefficient decreased to −0.334 and −0.28 after state anxiety and career satisfaction were introduced, respectively. Therefore, both state anxiety and career satisfaction acted as mediating factors for person–vocation fit and nominative commitment. From models 3, 8, and 9, the regression coefficient of CSE to normative commitment was 0.255 (p < 0.001) when the former was used as the independent variable. The regression coefficient became insignificant after state anxiety and career satisfaction was introduced, which indicates that the prediction of normative commitment by CSE was completely intermediated by state anxiety and career satisfaction.

**Table 5:** Stepwise regression of normative commitment

	**Model 1**	**Model 2**	**Model 3**	**Model 4**	**Model 5**	**Model 6**	**Model 7**	**Model 8**	**Model 9**
Personal variables									
Development unfitness		−0.416^***^				−0.334^***^	−0.28^***^		
Core self evaluations			0.255^**^					0.106	0.09
Anxiety				−0.388^***^		−0.285^**^		−0.336^***^	
Career satisfaction					0.489^***^		0.396^***^		0.456^***^
*R*^*2*^	0.114^**^	0.263^***^	0.168^**^	0.233^***^	0.331^***^	0.32^***^	0.389^***^	0.237^***^	0.333^***^
*△R*^*2*^	0.114^**^	0.145^***^	0.056^**^	0.118^***^	0.209^***^	0.203^***^	0.268^***^	0.127^***^	0.215^***^

Note: **, *p* < 0.01; ***, *p* < 0.001.

**Figure 1:**
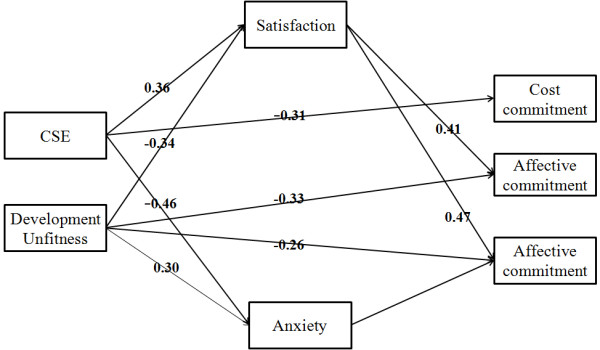
**The whole model representation.** Note: Factor loadings are standardized.

Based on above, we made a path analysis on the whole model, and the results were shown on Figure 1. The following four indices were used to evaluate the goodness of fit of the model: (a) Chi-square statistic (χ^2^), χ^2^/df, (b) the Standardized Root Mean Square Residual (SRMR), (c) the Root Mean Square Error of Approximation (RMSEA), and (d) the Comparative Fit Index (CFI). In this study, a model was considered to have a good fit if all the path coefficients were significant at the level of 0.05, SRMR was below 0.08, RMSEA was below 0.08, and CFI was 0.95 or more.

The results revealed the results showed that the model very good fit to the data, χ^2^ (8, N = 167) = 18.315, P = 0.019; RMSEA = 0.07; SRMR = 0.062; and CFI = 0.978. Hoelter Index was used to reinforce the overall fit provided by CFI. HOELTER (0.01) =182, revealed the largest sample size for which one would accept at 0.01 significance that the model is correct.

The mediating effect of job satisfaction and anxiety between CSE, DU and the three dimensions of career commitment was tested for a significance by adopted the Bootstrap estimation procedure in AMOS (a bootstrap sample of 1200 was specified). Table 6 shows the indirect effects and their associated 95% confidence intervals.

**Table 6:** Direct and indirect effects and 95% confidence intervals for the final model

**Model pathways**	**Estimated effect**	**95% CI**	
		**Lower bonds**	**Up bonds**
**Direct effect**			
CSE → Cost commitment	−0.312^*^	−0.449	−0.160
Development unfitness → Nominative commitment	−0.330^*^	−0.442	−0.220
Development unfitness → Affective commitment	−0.284^*^	−0.398	--0.159
**Indirect effect**			
Development unfitness → Satisfaction → Nominative commitment	−0.139^*^	−0.190	−0.063
Development unfitness → (Satisfaction, Anxiety) → Affective commitment	−0.203^*^	−0.301	−0.134
CSE → Satisfaction → Nominative commitment	0.149^*^	0.094	0.305
CSE → (Satisfaction, Anxiety) → Affective commitment	0.237^*^	0.239	0.509

^*^Empirical 95% confidence interval does not overlap with zero.

## Discussion

This research confirmed that the vocational values of medical university students did not fit their careers to some extent. Gaps between personal demands and job supply exist in all three dimensions, namely, self-development, basic security, and reputation and status. The unfitness was most prominent in the self-development dimension. According to the three-factor theory on person–environment fit [1,2], person–vocation fit also requires fits between similarities, personal demands, and career provisions, as well as vocational demands and personal capacity. Previous studies on person–vocation fit determined the suitability of individuals for their jobs by comparing their knowledge, skills, capacity, interests, working features, and personalities, which can reflect only the consistency or fitness between career requirements and personal capacities. Vocational values show the fitness between personal demands and career provisions, and to a certain degree, they reflect the values of individuals in choosing their careers. Vocational value–career fit shows the fitness between the personal demands of individuals and career provisions, and it reflects the consistency between the values of individuals and their careers to some extent. This study also somewhat expands the connotation of person–vocation fit.

This research proves that development unfitness in vocational value–career fit can predict the career commitment of individuals, which may be mediated by state anxiety and career satisfaction. Due to limited information and time, medical university students are not fully aware about the requirements and duties of doctors or nurses when they apply for medical universities and choosing medical careers. These students will only learn about the responsibilities they must shoulder as healthcare professionals upon entering medical universities. A medical career cannot completely satisfy medical university students in terms of their self-development, health care elements, reputation, and social status, which makes them highly concerned about their career and future. The career commitment of these students is reduced when their requirements are not satisfied by the medical field. Therefore, a wider gap between vocational values and medical careers intensifies the state anxiety of medical students, which will further reduce their satisfaction with and commitment to their selected careers.

CSE can significantly predict career commitment and career satisfaction, which is in line with the findings of Judge [4,5]. Both state anxiety and career satisfaction have mediating effects on core self-evaluation and career commitment. Medical university students who have low CSE usually develop a negative primary evaluation about their capacities and values, underestimate their own capacities, and amplify their present difficulties. These students may become highly anxious when they are faced with unfamiliar medical careers. As a result, their career satisfaction decreases. However, underestimations of their capacities and values will encourage these students to evaluate the costs that they have to bear when they leave their medical careers. In other words, these students must bear additional cost commitment upon leaving their chosen profession. Therefore, CSE is positively correlated with normative commitment and affective commitment, but is negatively correlated with cost commitment.

## Competing interests

The authors declare that they have no competing interests.

## Authors’ contributions

Conceived and designed the experiments: WX LZ JZ JP. Performed the experiments: WX LZ DM JZ JP. Analyzed the data: JZ JP. Contributed reagents/materials/analysis tools: WX QU YZ DM. Wrote the paper: WX LZ JZ JP. All authors read and approved the final manuscript.
